# Stimulus-Stimulus-Pairing to Reduce Stereotypies in Three Children with Autism during Movie Watching

**DOI:** 10.3390/bs11120165

**Published:** 2021-11-30

**Authors:** Marco Esposito, Maria Teresa Dipierro, Federica Mondani, Giulia Iurato, Paolo Mirizzi, Monica Mazza, Marco Valenti

**Affiliations:** 1 Department of Applied Clinical Sciences and Biotechnology, University of L’Aquila, 67100 L’Aquila, Italy; monica.mazza@univaq.it; 2 Autism Research and Treatment Centre, Una Breccia Nel Muro, 00168 Roma, Italy; mariateresa.dipierro@unabreccianelmuro.org (M.T.D.); federica.mondani@unabreccianelmuro.org (F.M.); 3 C.S.R. Sicilian Rehabilitation Consortium, 97016 Pozzallo, Italy; giuliaiurato94@gmail.com; 4 Department of Psychology, University of Bari, 70121 Bari, Italy; paolomirizzi@yahoo.it; 5 Regional Centre for Autism, Abruzzo Region Health System, 67100 L’Aquila, Italy; marco.valenti@univaq.it

**Keywords:** autism spectrum disorders, stimulus-stimulus pairing, challenging behaviors, stereotypies, applied behavior analysis

## Abstract

Autism spectrum disorders represent a challenge for professionals, who must include in their individualized educational interventions goals for core symptoms (social–communication and stereotypies/restricted interests) and comorbidities. The narrowness of interests and the high frequency of repetitive behaviors in children with autism often constitute an obstacle for learning and the quality of life, and for their caregivers as well. In the scientific literature, behavioral interventions based on both aversive and, less commonly, positive procedures have been implemented to reduce the frequency of stereotypies. The following study was carried out with the intention of replicating a Stimulus-Stimulus Pairing procedure applied by Nuzzolo-Gomez, Leonard, Ortiz, Rivera and Greer (2002) in order to reduce stereotypies in children. This procedure was applied to three children diagnosed with autism aged five, almost six and seven years, in order to reduce stereotypies when children watched movies. An A-B-A experimental design with three subjects was used for this research. The results showed a decrease in stereotypies in favor of appropriate behaviors.

## 1. Introduction

Children with Autisms Spectrum Disorders (ASD) are characterized by social communication deficit and a tendency to engage in a pattern of restricted and repetitive behaviours, including sensory anomalies, feeding issues and challenging behaviours [1]. Similarly, stereotypies can represent a serious obstacle to the development and learning of children. More than 72% of children diagnosed with ASD exhibit different forms of stereotypies [2], and their presence is inversely proportional to intellectual level [3]. Stereotypies are generally defined as a series of repetitive movements of the body [4] with no apparent adaptive significance, such as body swaying, mouth grimaces, or complex hand and finger movements [5]. Greer, Becker, Saxe and Mirabella [6] have provided a further definition of stereotypies as cycles of repetitive movements that have no apparent consequences, beyond the movement itself, for the individual who is emitting them. The original conceptualization of stereotypies as a response aimed at increasing or decreasing the level of environmental stimulation has been enriched by the most recent research, which has instead highlighted its multiple determinants, including positive sensory reinforcement, negative sensory reinforcement, positive social reinforcement and negative social reinforcement. In recent decades, several studies have also been conducted with the aim of preventing or correcting stereotypies [7], mainly through the use of aversive procedures such as punishment [8,9], reprimand [10], physical containment [9], overcorrection [11], timeout [12,13] and in some cases even electroshock [8,10]. These procedures have for years been considered the most effective in reducing stereotypies; however, they are effective only for short periods of time following their implementation [14], even without considering the associated ethical and legal implications [15]. Ahearn, Clarck, and MacDonald [16] described the use of a form of response blocking called Response Interruption and Redirection (RIRD), for the reduction of vocal stereotypies [17]. Although less prevalent in the literature, several positive approaches have also been successfully adopted to decrease stereotyped behaviors, such as differential reinforcement of other behaviors or DROs [18] and the teaching of functional play [19,20]. However, although some comparative studies have shown that DRO was slightly less effective than punitive procedures in reducing stereotypies [11,18] and that functional play was a valid competitive behavior with self-stimulation, in both cases there was no significant evidence of generalization and maintenance of the results obtained. Nevertheless, Eason, White and Newsom [21] have shown that the teaching of functional play determines a decrease in motor stereotypies [22] through the delivery of extrinsic positive reinforcers. Likewise, other authors have applied the principles of respondent conditioning to tackle the problem of stereotypies. Greer et al. [6] and Nuzzolo-Gomez et al. [14] used a conditioning procedure to teach students to select books or toys as their favorite activities, replacing stereotypies, using tokens and rewards as conditioned reinforcers. Recently, Tsai and Greer [23] have studied the effects of conditioning on book reading as a reinforcement activity. Specifically, the results of the research conducted by Nuzzolo-Gomez et al. [14] showed a reduction of stereotypies through the conditioning of functional play, suggesting the hypothesis that stereotypies also have a playful function and can be replaced precisely by functional play behavior. At the same time, the study conducted by Tsai and Greer [23] showed how a book conditioning procedure led to a reduction in the number of learning units necessary to satisfy the criterion of accuracy in reading tests. Hence, through conditioning it was possible to promote the learning of reading. For many people with disabilities, conditioning procedures are necessary in order to condition important stimuli for development and learning, for example, functional play and appropriate behaviors. One of the most widely used methods is stimulus-stimulus pairing based on respondent conditioning.

### 1.1. Respondent Conditioning

Respondent conditioning occurs when an organism responds to a new event based on a history of association with a biologically important stimulus. The Russian physiologist Ivan Pavlov discovered this form of conditioning at the turn of the century. He showed that dogs salivated when food was placed in their mouths. This relation between the food stimulus and salivation is called a reflex and occurs because of the animals’ biological history. When Pavlov rang a bell just before feeding the dog, it began to salivate at the sound of the bell. In this way, new features (sound of the bell) controlled the dog’s respondent behavior (salivation). Thus, presenting stimuli together in time (typically, a conditioned stimulus followed by an unconditioned stimulus) is the procedure for respondent conditioning. If a conditioned stimulus (CS) comes to regulate the occurrence of a conditioned response (CR), respondent conditioning occurs (for more information, please see www.scienceofbehavior.com, accessed on 13 September 2021).

### 1.2. Definition of Stimulus-Stimulus Pairing

Stimulus-Stimulus Pairing (SSP) has been used to condition vowel sounds in students with a limited verbal repertoire [24] and to expand the variety of reinforcements [6,14,25,26,27]. The process of respondent conditioning occurs by the contingent presentation of two or more stimuli concurrently. The exhibition of the stimuli can occur at the exact same moment, or one stimulus can precede the other stimulus in time; however, the exhibition of the second stimulus generally must quickly follow the onset of the first stimulus. It can be defined as a procedure through which the repeated associations between two stimuli allow the neutral stimulus to acquire the same properties as the reinforcing stimulus to which it has been associated [28]. Concerning scientific literature on SSP applied in order to reduce stereotypies in children with ASD, several previous authors are discussed here. Two experiments were conducted with four students with autism in an attempt to teach children with autism to prefer books or toys over stereotypy or passivity [14]. The association between toys or books and conditioned reinforcers was applied to increase use of books or toys, decreasing stereotypy and passivity. A relevant experiment involved a multiple baseline across three students. Students who exhibited frequent rates of stereotypy in the baseline had significantly fewer intervals of stereotypy after toys were conditioned as reinforcers, and toy play increased for all the students. Similarly, the SSP procedure was applied to teach two preschool children with autism in order to facilitate toy play and replace stereotypies [20]. The dependent variables were the number of correct/incorrect responses and intervals of stereotypies emitted during sessions. The conditioning procedure demonstrated to be an effective intervention in increasing functional toy play and decreasing stereotypies. These findings support the choice of positive interventions to improve functional behaviors over challenging ones for children with autism during free time. In addition to adding evidence regarding respondent conditioning and its relationship with functional behaviors/stereotypies, the present study was conducted in order to reduce stereotypies in three subjects with ASD while watching movies, through a procedure involving SSP for response blocking of stereotypies.

## 2. Materials and Methods

### 2.1. Participants

The participants, aged 70 months on average (DS = 12.5), were selected on the basis of a diagnosis of ASD made by experienced clinicians based on the criteria of the Diagnostic and Statistical Manual of Mental Disorders (DSM-5) and assessments by means of standardized diagnostic instruments: the Autism Diagnostic Observation Schedule–Generic [29] ADOS-G, the Autism Diagnostic Interview–Revised [30] ADI-R, the Griffiths Mental Developmental Scale–Extend Revised [31] GMDS-ER, and the Vineland Adaptive Behavior Scales [32] VABS. Children were not recruited if they had seizures, were eight years of age or older, or had additional medical diagnoses (e.g., genetic syndromes). All the children showed restricted interests and severe stereotypies, decreasing learning and functional play. All the children were Italian, and for two years before the current training had been following comprehensive ABA treatment [33] that included 1:1 therapy with a behavioral therapist (with half of this time spent in quasi-natural settings) divided between discrete trial teaching (DTT) and naturalistic environment teaching, or NET [34] at the research and treatment centre “Una Breccia nel muro”, through a modification of a previous clinical model [35]. The therapists and supervisors had a minimum of a master’s degree in applied behavior analysis and two years of experience in treatment of children with ASD. Each child’s skills, strengths, and deficits were also evaluated on the basis of their performance in the Assessment of Basic Language and Learning Skills [36] carried out by the assigned supervisor. The ABLLS was used both to identify learning goals and to control either progress or regression during the standard treatment. Additionally, the intervention comprised direct supervision (1 h a week) by an expert professional to ensure the reliability of teaching procedures and parent inclusion (parents spent 2 h a week in the therapy room with clinical staff). The caregiver observed the child’s therapy weekly with the presence of the staff. The individualized educational plan for the children involved preschool goals regarding communication, attention, imitation, social play, gross and fine motor skills, receptive and expressive language, autonomy, and management of problem behavior. After two years of the standard therapy described above, treatment regarding stimulus-stimulus pairing was begun. The research was conducted with the prior written consent of both parents and with the approval of the host institution either to participate in the research program or to publish the content of the current intervention. The study was submitted having followed the norms required by the Code of Ethics of Research in Psychology (www.aipass.org/node/11560, accessed on 14 September 2021) and Child Development (www.srcd.org/about-us/ethical, accessed on 14 September 2021) as well as the BACB’s ethics requirements (www.bacb.com/ethics-information, accessed on 14 September 2021). Furthermore, certified behavior analysts, as supervisors of the participants, informed their caregivers in understandable language about the nature of the research and its publication; about significant factors that could influence their willingness to participate; answered any other questions participants had about the research; and informed them that they were free to depart from the research at any time without penalty [15].

Participant A was a seven-year-old boy with a diagnosis of ASD and mental retardation who attended the first year of primary school two weeks a month for four hours a day with a shadow teacher and school educator. The child’s diagnosis was established by an external child neuropsychiatry equip through a protocol that involved the ADOS, ADI-R and Griffith Scales. At school, this child worked separately from the class group and carried out speech therapy and neuro-psychomotricity sessions at school assigned by public health service. The child communicated mainly using single words, responded to simple instructions, recognized at least one hundred objects and named about fifty images and objects before the training. He also walked on his toes, showed motor stereotypies (clapping, belly movements, swinging on a chair and hand flapping) that limited the learning of new skills, functional play and daily life activities. The child had been attending a behavioral treatment for two alternating weeks for a month for a total of 40 h a month. 

Participant B was a five-year-old boy diagnosed with ASD who attended kindergarten every morning with a shadow teacher and an ABA tutor who spent six hours with him. The child’s diagnosis was provided by an external child neuropsychiatry equip through a protocol of ADOS, ADI-R, and Psychoeducational Profile PEP-3 [37]. The child at school responded to all the activities within the class, communicated mainly using single words and occasionally simple sentences containing the verb and the object complement, responded to simple instructions, recognized many objects and images of different semantic categories, naming about one hundred between objects and images. In addition, the child showed restricted interests and motor stereotypies (rubbing his face, swinging wires or hair, moving his hands and objects near his eyes) that limited functional play and compliance with adults’ requests during the daily life. This child had been following a behavioral intervention at home and at school (18 h a week; six at school and twelve at home) supervised by a consultant.

Participant C was a five and a half year old child diagnosed with ASD, as established by the pediatric neuropsychiatry equip, through the administration of the ADOS, ADI-R and Griffith scales. The child was able to match images and objects. He was able to imitate movements with objects or even lines or simply drawings, recognize and discriminate objects and images, and play in turns displaying symbolic plays. He used an electronic Augmentative Alterative Communication (CAA) system, vocalizing the selected icons and adding images of new categories. This system was installed on the child’s personal tablet, and he was able to form phrases with object + adjective and to answer simple social questions. The child displayed a variety of interests and responded to social reinforcement. However, he exhibited numerous motor stereotypies that limited his ability to respond to adult’s requests in his daily life. He had been following a behavioral weekly intervention involving 6 h of treatment per week, divided into three sessions of 2 h. The therapy included one hour of one-to-one therapy and one hour of therapy in the playroom with other children.

### 2.2. Settings and Materials

The training of participant A was conducted at the centre in a room where there was a table and a shelf with play items. The training of the participant B was conducted at the child’s home, in a small room containing a bed, a wardrobe, a shelf with visible play items and a toy box containing games and materials used during the therapy sessions. The training of the participant C was conducted at the centre in a room used for standard therapy, in which there was a table, a chair and a computer used for watching cartoons. The current program comprised an assessment of the efficacy of the training SSP based on a repeated baseline (pre and post probes with follow-up). Each weekly session of training lasted 400 seconds and was conducted with a 1:1 ratio between the tutor and participant. The participants correspondingly needed 48, 22, 9 sessions, respectively, before acquiring the mastered criterion of the training. The materials that were used for the probes and stimulus-stimulus pairing sessions included a table, a chair and a computer. During the procedure, the children were seated on the chair, and the tutor was placed behind them in order to be a moderate presence but at the same time able to deliver reinforcers. The therapists used a pen for data recording, a data sheet, and a timer to measure the stereotypies of children occurred during the training, following two categories of intervals: the SSP (respondent conditioning) and observation intervals (see Procedure for more details). 

### 2.3. Experimental Design

The study employed an A-B-A experimental design on three single subjects with follow-up (Table 1). Regarding participants A and B, the follow-up was performed after one month, while for participant C two follow-ups were conducted, one after two weeks and another after four weeks from the intervention. Concerning the experimental design, phase A represents the baseline, while Phase B represents the implementation of the stimulus-stimulus pairing procedure (training); finally, the last phase represents the observation conducted post-probe.

### 2.4. Procedure

Concerning the training, we operationally defined the behaviors that were recorded during the probes and during the intervention and coded appropriate behaviors shown by children with a plus sign (e.g., watching the video sitting with hands on the table) and inappropriate ones (e.g., body stereotypies) with a minus sign 

A preference assessment was also conducted, and the first reinforcers identified in the hierarchy were used for the training. We selected a specific reinforcer for the training and deprived the children of that reinforcement for the rest of the time (i.e., time out of the training). We performed two probes of five minutes (A), each divided into 60 five-second intervals, for data recording. Each participant was asked to sit in front of the table on which a computer displayed cartoons. During the probes the experimenter did not provide any correction and did not deliver reinforcers, merely recorded the data. During the intervention phase (B), the experimenter was positioned behind the child in order to easily deliver the reinforcer and be less invasive. Each intervention session included 20 learning units, which consisted of ten seconds of pairing alternating with ten seconds comprising only behavior observation (Test Condition). During the pairing intervals, no instructions were given, motor stereotypies were blocked, and physical guidance was provided to place the hands of the children on the table, redirecting the child to the computer. After these behavioral strategies, the reinforcer was delivered. During the test intervals the experimenter only recorded data. The mastery criterion was set at 90% of correct intervals for two consecutive sessions or 100% of correct intervals for a single session. After the acquisition of the criterion, two post-probes were carried out in the same way as the pre-probes. After one month from the end of the intervention, we conducted a follow-up to evaluate the appropriate behaviors and the reduction of stereotypies. The follow-up was conducted in the same way as the probes. No other intervention on the reduction of stereotypies was introduced during the training. The dependent variables in this study were the percentage of the number of intervals in which appropriate behavior was emitted and the percentage of the number of intervals in which stereotypies occurred during the test conditions. The independent variable introduced was represented by a stimulus-stimulus pairing procedure conditioning appropriate behavior during the movie watching.

### 2.5. Data Collection

During the probes, data were recorded by the experimenter using a data sheet in which the intervals were already divided in sections of five minutes. We used a pencil and paper procedure with a mobile application that provided an alert whenever an interval concluded (10 s). For each interval the data were recorded using a positive sign (+) for a correct answer and a negative sign (−) to encode inappropriate behaviors, stereotypies, and passivity. The data were plotted on a graph showing the percentages of the number of intervals during which the appropriate behavior was exhibited and the number of intervals in which inappropriate behaviors occurred. During the intervention, the experimenter collected data concerning 20 test conditions. Specifically, each learning unit included 10 s of pairing alternating with 10 s of testing in which the experimenter simply collected the data without interacting with the child. After completing 20 pairing and testing intervals, the number of intervals in both those with appropriate behavior and those with inappropriate behaviors were plotted on a percentage graph. In both probes and during the intervention, whole-interval recording (WIR) was used for appropriate behavior. That is, the behavior was coded using the positive sign (+) only if it occurred throughout the interval (10 s). Regarding the inappropriate behaviors (stereotypies and passivity), partial interval recording was used. For example, it was coded with the negative sign (−) if the behaviors described above occurred in a partial time interval within 10 s.

## 3. Results

**Figure 1:**
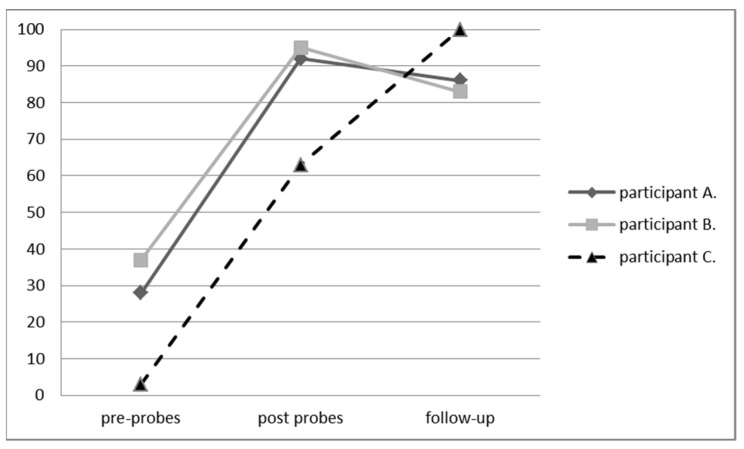
Percentages of positive behavior intervals of the three participants. Probes: 5 min divided in 10 s intervals; follow-up 30 days after training; number of sessions to acquire mastered criterion 48, 22, 9, respectively, for the three participants A, B, and C. The analysis of frequency differences applying a chi-square statistic is 13.372. The *p*-value is 0.001. The results are significant at *p* < 0.05. Data analysis was performed using the R package Version 1.15.

Starting from the descriptive statistics collected on the percentages of positive intervals (appropriate behavior) and negative intervals (stereotypies) during the probe and training session, participant A in the two pre-probes showed an average of 28.3% positive intervals (DS = 2.36), and in the post-probes showed 91.67% positive intervals (DS = 2.36), showing a significant increase in functional behaviors and a decrease in stereotypies. The training mastery criterion was reached after 48 sessions. Likewise, participant B in the two pre-probes showed an average of 36.67% positive intervals (DS = 4.71), and in the post-probes showed 95% positive intervals (DS = 2.36), showing an increase in functional behavior and a decrease in stereotypies. The training mastery criterion was reached after 22 sessions. Finally, participant C in the two pre-probes showed on average 3.5% positive intervals (DS = NA), and in the post-probes showed 63.5% (DS = NA) positive intervals. The training mastery criterion was reached after nine sessions of training. After 30 days from the end of intervention, a follow-up was conducted. Participant A displayed 86.6% positive intervals, participant B 83.3% positive intervals, and participant C 100% positive intervals (please see the Figure 1).

## 4. Discussion

The current study aimed to gather information concerning the efficacy of respondent conditioning (stimulus-stimulus pairing) on the stereotypies of three children with autism. Likewise, we have replicated the researches of Nuzzolo-Gomez, where edible and social reinforcers were delivered while children observed books and played with toys. Specifically, the reinforcing stimuli and neutral stimuli (such as books and toys) were associated during free play sessions. Hence, in the case that the neutral stimulus became a conditioned reinforcement, it was consequently selected more often by the participant, determining the reduction of the stereotypies. In this study the stimulus-stimulus pairing procedure was not adopted to condition neutral stimuli, as in previous researches, but rather to reduce the dysfunctional use of a stimulus that was already a conditioned reinforcer, such as movies. Therefore, the data obtained by training show the efficacy of SSP along with response blocking in reducing the stereotypies of the children enrolled. In fact, only a few training sessions were generally needed to determine an increase in appropriate behaviors and a decrease in motor stereotypies. Going by the number of sessions needed by the participants in order to acquire the mastery criterion of training, we also ought to consider the clinical differences of the children at their respective baselines, which can have an influence on the results, as well as the previous behavioral interventions they have been following (i.e., low-intensity treatment). For example, participant A reported a double diagnosis of ASD and mental retardation, with 48 sessions to reach the mastery criterion. As a result, the severity of symptoms could have an impact when using outcomes as guidelines in behavioral intervention reports. Nevertheless, the current procedure is based on manualized reinforcement schedules, referring to respondent and operant conditioning. Consequently, the paper shows that these behavioral applications are corrected on the basis of efficacy and evidence [38]. Furthermore, these results also corroborate the thesis according to which positive procedures for this type of treatment are preferable to punitive ones, as unlike interventions based on punishment (positive or negative), this type of intervention combines the need to reduce inappropriate behaviors by teaching alternative functional and more socially acceptable forms of behavior. It should be considered that during the entire period of treatment outside the rehabilitation center the children were not exposed to the movies included in the procedure, and they were deprived of the reinforcements used for conditioning; the limited use of reinforcement may have contributed to maximizing the effectiveness of the training. Moreover, the research was conducted for a continuous period of time, without suspensions, maintaining the same staff for all of the children. As a result, this stability may have reduced some threats to the internal validity of the design, such as maturation and learning history. The implications of the use of stimulus-stimulus pairing, specifically on reducing stereotypies and other challenging behaviors in children with autism are confirmed by previous evidences [14,20]. Mainly, we would like to discuss some aspects of the application of behavioral models for children with autism. Firstly, a therapy based on ABA procedures comprises a direct or indirect stimulus preference assessment in order to rank potential reinforcements. However, in the case of severe behaviors and restricted interests, behavioral analysts can recommend clinical staff expand greater variety or frequency regarding the interests of children. Consequently, SSP could represent a clinical choice in order to improve the capability of external reinforcement, even for discrete teaching applications and natural interventions. On the other hand, these procedures based on respondent conditioning allow a reduction in stereotypies and other challenging behaviors during functional play. These considerations could help to better highlight the contributions of the current study in the existing body of work on interventions addressing stereotypies, and provide suggestions to researchers, clinicians and students. Additionally, interventions including SSP aim to meliorate some core symptoms of children, such as restricted interest and stereotypies, with respect to treatment including other skills such as motor, academic and cognitive skills. Accordingly, the clinical choice to manage behaviors which limit learning as a priority should be addressed. 

Concerning the duration of intervals (gradually longer) where appropriate behaviors occurred, we can hypothesize that the data that emerged during the application of the procedure are the result of a generalization process, without prior learning for those specific conditions. Generally, in typical development the generalization is inherent in the learning itself where it occurs naturally. It is possible to explain the generalization as a learned behavior in response to different antecedents from the original with shared characteristics. Thanks to generalization, a person’s ability to act in new situations similar to others becomes natural. Likewise, the participants in this study, in fact, met the criteria for mastering the procedure in adequate time. Increasing appropriate behaviors in place of stereotypies while watching the videos has allowed these children’s parents to be able to manage their free time in a more functional and adequate way, helping to improve daily family organization, especially at home. 

However, the present study shows some limitations. First, the absence of agreement between observers (IOA); the work organization of the center provided a 1:1 ratio between the therapist and the child and clinical staff. Additionally, at the time of training, the treatment center was not able to provide any other employees. Another limitation is given by the fact that while follow-up was conducted for the three participants, generalization of their learning in other settings was not verified in the absence of adults or in the presence of people other than the therapist. This limitation of the conditions of generalization across contexts and people should be better assessed in future studies through cross-sectional follow-ups, in order to evaluate the effectiveness of the stimulus-stimulus pairing independent of the context in which it is implemented and of the people who implement the procedure, which would be able to demonstrate clinical as well as procedural efficacy. On the other hand, in order to ameliorate the research methodology of the single case studies, it is possible to apply a multiple baseline design across participants, controlling and manipulating the independent variable. Moreover, concerning the research design used, the removal of the independent variable after the intervention phase does not determine the return to the starting condition; this constitutes a procedural limit. However, it can be considered a clinically desirable result. It was not possible to demonstrate the existence of a functional relationship between the dependent variable and the independent variable; however, in the removal phase of the intervention, the participants showed a high percentage of ranges of appropriate behaviors. Future developments of this research could provide a return to the baseline after the teaching phase, formalizing an unpairing process in order to evaluate both the effectiveness of the functional relationship in the intervention phase and the number of sessions necessary to implement an unpairing process when compared with the number of sessions required to implement it. Some ethical limitations, however, should be considered in this case, above all due to the risk of a failure to re-condition the stimulus itself. Having more stimuli could be helpful in bypassing this problem.

Finally, despite the limitations described above and the usefulness of extending the research, this work confirms the results reported in the literature, namely that it is possible and preferable to select positive procedures for the reduction of problematic behaviors in order to obtain equally effective results. Essentially, applications based on SSP can help professionals to address the restricted interests and stereotypies of children with autism in order to expand their tailored educational programs and settings. These opportunities could serve to reduce core symptoms, with the purpose of allowing more reinforcements and collaboration in learning in both discrete trial teaching and in natural conditions.

## 5. Conclusions

Currently, children with autism spectrum disorders represent a professional challenge since they show deficiencies in social communication and stereotypies/restricted interests and comorbidities (epilepsy, mental retardation, language deficits, sleep/anxiety disorders and food selectivity). Behavioral interventions applied in either early childhood or in adolescence has demonstrated efficacy in increasing skills and in reducing the types of challenging behaviors which limit learning. Clinicians and behavioral analysts can refer to manualized and research-based treatments before the implementation of an individualized behavioral program, either comprehensive or focused on people with autism. The following study, starting from the main principles of behavioral science (that is, respondent conditioning), was carried out with the intention of replicating a Stimulus–Stimulus Pairing procedure to reduce stereotypies in three children with autism. The current results show a decrease in stereotypies in favor of appropriate behaviors during movie watching. Likewise, we have discussed the ways that these conditioning procedures can have an impact in other educational programs involving a variety of interests in order to ameliorate functional play using more reinforcements for both structured and natural teaching. Finally, since the narrowness of interests and the high frequency of repetitive behaviors in children with autism often constitute an obstacle for learning, reducing the quality of life of their caregivers, these behavioral programs respond to a major social need, ameliorating learning opportunities and family routines.

## Figures and Tables

**Table 1:** Example of experimental design applied.

Experimental Design (All Three Participants Started at Different Times)			
A	B	A	A
* two pre-probes	** SSP	* two post-probes	follow-up
Baseline	Training	Baseline	Baseline

Note. * Each lasted 5 min; ** number of sessions: A (48), B (22), C (9).

## Data Availability

Data regarding the treatment can be gathered by sending a request to info@unabreccianelmuro.org (Autism research and treatment center una breccia nel muro, Rome).
